# Use of Wearable Sensors to Assess Fall Risk in Neurological Disorders: Systematic Review

**DOI:** 10.2196/67265

**Published:** 2025-08-18

**Authors:** Mirjam Bonanno, Augusto Ielo, Paolo De Pasquale, Antonio Celesti, Alessandro Marco De Nunzio, Angelo Quartarone, Rocco Salvatore Calabrò

**Affiliations:** 1 IRCCS Centro Neurolesi Bonino-Pulejo Messina Italy; 2 Department of Mathematics, Computer Science, Physics and Earth Science University of Messina Messina Italy; 3 Department of Health LUNEX University of Applied Sciences Differdange Luxembourg

**Keywords:** fall risk assessment, wearable sensors, neurological disorders, neurorehabilitation, artificial intelligence, AI

## Abstract

**Background:**

Assessing fall risk, especially in individuals with neurological disorders, is essential to prevent hospitalization, hypomobility, and reduced functional independence. Wearable sensors are increasingly used in neurorehabilitation, as they enable unsupervised fall risk assessment by providing continuous monitoring during daily functional tasks, thereby offering a reflection of the individual’s real-world fall risk.

**Objective:**

We systematically reviewed the literature on reliable biomechanical gait parameters detected with wearable sensors to assess fall risk in neurological disorders, focusing on patients with Parkinson disease, multiple sclerosis, stroke, or Alzheimer disease. In addition, we examined the latest advancements in wearable sensor technology, including best practices for device placement as well as data processing and analysis.

**Methods:**

We conducted a comprehensive systematic search for relevant peer-reviewed articles published up to April 18, 2025, using PubMed, Web of Science, Embase, and IEEE Xplore, which are the most used databases in the fields of health and bioengineering.

**Results:**

The 19 included studies involved 2630 patients with neurological disorders, including 226 (8.59%) with multiple sclerosis (n=7, 37% studies), 2305 (87.64%) with Parkinson disease (n=8, 53% studies), 51 (1.94%) with stroke (n=3, 16% studies), and 48 (1.83%) with Alzheimer disease or cognitive impairment (n=1, 5% study).

**Conclusions:**

This review highlights the role of wearable technologies in assessing fall risk in patients with neurological disorders. Although the included studies showed variation in methods and a focus on technology over clinical context, the lack of standardization reflects ongoing advancements, which may be seen as a strength.

**Trial Registration:**

PROSPERO CRD42023463944; https://www.crd.york.ac.uk/PROSPERO/view/CRD42023463944

## Introduction

### Background

Neurological disorders, such as Parkinson disease (PD), multiple sclerosis (MS), Alzheimer disease (AD), and stroke, are significant and growing global health concerns due to their high prevalence. A recent meta-analysis reported a global pooled prevalence of PD of 1.51 cases per 1000 individuals, with higher rates observed among male individuals and a significant increase over the past 4 decades [1]. Similarly, MS currently affects approximately 2.8 million people globally, with rising prevalence and incidence trends observed worldwide [2]. In Europe, the prevalence of AD has been estimated at 5.05% (95% CI 4.73%-5.39%) [3]. The incidence of clinically diagnosed AD per 1000 person-years increases markedly with age: 3.4 for individuals aged 65 to 74 years, 13.8 for those aged 75 to 84 years, and 35.8 for those aged ≥85 years [4,5]. Regarding cerebrovascular impairments, stroke continues to be a leading cause of death and disability, with >12 million new cases per year and >100 million survivors of stroke worldwide [6].

These conditions often lead to alterations in gait and balance, increasing the risk of falls. This risk is especially pronounced in older adults and individuals with neurological impairments. Approximately one-third of adults aged >65 years experience a fall each year [7], and this rate rises to nearly 50% among those aged >80 years [8]. Consequently, falls in this patient population considered fragile can worsen quality of life due to hospitalization, hypomobility, and reduced functional independence [9]. Fall-related injuries not only entail substantial medical costs but also determine patients’ mortality risk. Among neurological disorders, PD, MS, cerebrovascular accidents, and AD are among the most common, causing significant motor disability. It is noteworthy that these patients with neurological disorders are considered recurrent fallers due to their gait features. In particular, it is estimated that 39% of patients with PD experience falls, while 40% to 60% of patients with MS experience recurrent falls [10]. Similarly, patients with stroke are at high risk for falls, with fall frequencies ranging from 27% to 39% in rehabilitation hospitals [10]. Furthermore, older adults with AD are more than twice as likely to experience a fall compared to cognitively healthy peers, with rates ranging from 60% to 80% [11]. However, each neurological disorder is characterized by considerable heterogeneity in gait disturbances due to differences in underlying etiology [12]; for example, neurodegenerative disorders (such as PD, MS, and AD) are associated with distinct gait patterns, characterized by reduced gait speed, shorter step length, and increased muscle weakness [12]. In addition, ambulation in patients with PD is characterized by shorter steps, loss of dissociated arm and trunk movements during gait, and postural instability [13]. By contrast, patients with MS manifest pathological gait features related to spasticity, ataxia, muscle weakness, and sensory and proprioceptive deficits, all of which contribute to progressive disability [14]. AD independently increases fall risk due to its associated impairments in judgment, gait, visuospatial perception, and hazard recognition [15]. Differently from neurodegenerative disorders, patients with stroke are characterized by a marked spatiotemporal asymmetry between steps, which substantially increases fall risk [16]. For these reasons, gait and balance assessment in patients with neurological disorders is essential in all phases of the rehabilitation process.

To this aim, clinical scales (ie, the timed up and go [TUG] test, the Tinetti Scale, the Falls Efficacy Scale–International, and the Berg Balance Scale) as well as innovative technologies, including wearable sensors and nonwearable devices, are used. Specifically, wearable sensors are growing in popularity in both clinical and research contexts due to their suitable features [17]: because of their small size, affordability, and user-friendly nature, wearable sensors represent an optimal solution for health monitoring [18,19]. Unlike traditional laboratory-based monitoring systems, wearable sensors can facilitate the continuous monitoring of motor activity by capturing physiological data during everyday routines [20]. In addition, fall risk assessments with laboratory-based systems are generally performed in supervised conditions, in which the behavior adopted by participants may not be representative of the one adopted in everyday life—during the experimental tasks, the participants might be attempting their “best effort” [21]. Generally, falls occur in unpredictable situations during everyday activities [22]. In this context, wearable sensors enable unsupervised fall risk assessment by providing continuous monitoring during daily functional tasks, thereby offering a reflection of the individual’s real-world fall risk [20,21,23].

Thus, interest in wearable sensors has been increasing with regard to monitoring fall risk among older adults [21]. Among the prevalent wearable technologies used for health monitoring are inertial measurement units (IMUs), which gather data from accelerometers and gyroscopes. IMUs can be strategically positioned on the body to capture motion data for subsequent analysis, integration, and interpretation [24]. Gait characteristics that can be analyzed include stride length, stance-to-swing phase ratio, and various other spatiotemporal gait parameters [21,25,26]. In addition, data from inertial sensors collected during walking can be used to investigate upper body dynamics through nonlinear analysis techniques, such as recurrence quantification analysis [27]. Some researchers have used inertial sensor data to detect balance alterations by analyzing sway speed in the anterior-posterior and medial-lateral directions of the body’s center of mass projection path in patients with AD [28].

Other wearable sensors include insole-based devices, which have been developed for fall risk assessment and detection. These devices are embedded in the shoe sole to capture data on plantar pressure distribution [21]. The features extracted from these devices are used to objectively quantify the risk of falling in individuals with neurological disorders. However, the selection of the most reliable biomechanical gait parameters related to fall risk remains an open question [21]. In addition, the information acquired from wearable sensors, along with data processing, data fusion, and analysis techniques such as machine learning (ML) models or manual data analysis, is used to extract meaningful insights from the investigated parameters [29].

To this aim, some studies have investigated the role of deep learning (DL) in the context of neurorehabilitation. The DL-based approach can be used to extract data on stride-specific gait parameters from IMUs [30]. This approach could be useful in the field of telemedicine as a potential in-home gait monitoring tool, given that alterations in gait function can serve as a biomarker for predicting fall risk.

### Objectives

In this context, our first objective was to systematically review the literature on the most reliable biomechanical gait parameters, as detected by wearable sensors, for evaluating fall risk in individuals with neurological disorders. Therefore, we selected studies that involved patients with PD, MS, AD, or stroke, as these conditions collectively represent a significant proportion of neurological disorders worldwide and are associated with substantial gait impairment and a high risk of falls. Our second objective was to investigate existing literature on wearable sensor technology, including best practices for device placement as well as data processing and analysis.

## Methods

### Overview

We conducted a comprehensive systematic review to explore the existing evidence on fall risk assessment in patients with neurological disorders. We summarized the results of all published studies in accordance with the PRISMA (Preferred Reporting Items for Systematic Reviews and Meta-Analyses) guidelines [31]. The PRISMA checklist is available in Multimedia Appendix 1. The protocol has been registered with PROSPERO (CRD42023463944).

### Population, Exposure, Comparator, Outcomes Question

We used the population, exposure, comparator, outcomes model to define the search strategy [32] (Table 1). The population, exposure, comparator, outcomes framework was applied to address the following research question: “what are the most reliable biomechanical gait parameters detected with wearable sensors to assess fall risk in neurological disorders?”

**Table 1:** Population, exposure, comparator, outcomes (PECO) framework for included studies.

PECO elements	Definition	Search terms
Population	Patients with neurological disorders, including cerebrovascular diseases, Parkinson disease, Alzheimer disease, and multiple sclerosis	“stroke” OR “Parkinson’s disease” OR “multiple sclerosis” OR “Alzheimer”
Exposure	Fall risk assessment using wearable sensors or gait analysis using wearable sensors	“wearable”
Comparator	Studies including a comparison with healthy controls	—^a^
Outcomes	Biomechanical gait parameters related to fall risk	“fall”

^a^Not applicable.

### Search Strategy and Eligibility Criteria

We conducted a systematic search for all peer-reviewed articles published up to April 18, 2025, using PubMed, Web of Science, Embase, and IEEE Xplore, which are the most used databases in the fields of health and bioengineering. The full search queries are provided in Multimedia Appendix 2. We included studies involving adults (aged ≥18 y) with stroke, PD, MS, or AD. Specifically, the inclusion criteria were as follows: (1) adult patients with cerebrovascular impairments, PD, MS, or AD; (2) fall risk assessment using wearable sensors; (3) articles written in English; and (4) published in a peer-reviewed journal. We excluded articles describing theoretical models, methodological approaches, algorithms, and basic technical descriptions. We also excluded animal studies, conference proceedings and reviews, studies involving children, and case reports. The list of articles was refined for relevance, revised, and summarized, with the key topics identified from the summaries based on the inclusion and exclusion criteria. Considering the limited literature available, we included the following study designs in the qualitative synthesis: (1) randomized controlled trials, (2) observational studies, (3) cross-sectional studies, (4) case-control studies, and (5) cohort studies. All search results were imported into Rayyan (Rayyan Systems Inc) [33], a web application designed to facilitate the systematic review process, and screened by 2 blinded reviewers (AI and MB). Each reviewer independently screened the articles to ensure consistent application of the inclusion and exclusion criteria. After screening titles and abstracts, blinding was removed. In cases of disagreement, the 2 reviewers discussed the inclusion or exclusion of the undecided articles to reach consensus.

### Data Extraction and Analysis

After full-text selection, data from the included studies were extracted and organized in a spreadsheet. Information was summarized using the following fields: assigned ID number, study title, year of publication or presentation and first author, study aims and design, study duration, recruitment method and setting, inclusion and exclusion criteria, use of a control group, use of wearable sensors, informed consent, conflicts of interest and funding, type of intervention or evaluation and control, number of participants, baseline characteristics, intervention setting, types of biomechanical parameters related to fall risk, results, and key conclusions. Data extraction was performed independently and in a blinded manner by 2 reviewers. In cases of disagreement, a third reviewer was consulted to resolve discrepancies.

### Risk of Bias

The risk of bias in the included studies was assessed using the National Heart, Lung, and Blood Institute Quality Assessment Tool for Observational Cohort and Cross-Sectional Studies [34]. This tool comprises 14 questions, with each item rated as “yes,” “no,” “cannot determine,” “not reported,” or “not applicable.” On the basis of the responses, the overall quality of each study was categorized as good, fair, or poor. The risk of bias was assessed independently and in a blinded manner by 2 reviewers. In cases of disagreement, a third reviewer was consulted to resolve discrepancies.

## Results

### Overview

**Figure 1:**
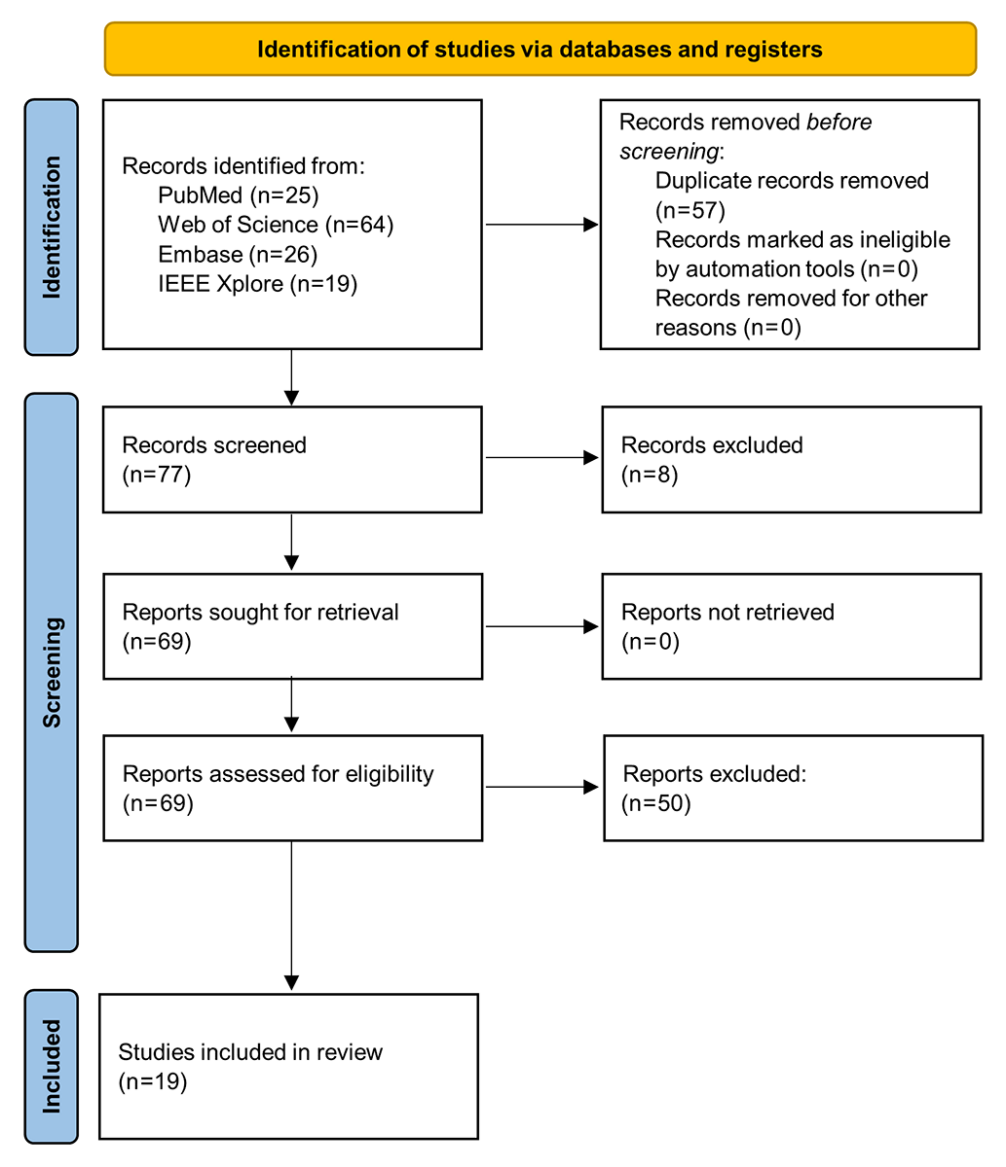
PRISMA (Preferred Reporting Items for Systematic Reviews and Meta-Analyses) flow diagram showing the number of studies identified, screened, assessed for eligibility, and included in the final analysis.

A total of 134 articles were identified through database search (PubMed: n=25, 18.7%; Web of Science: n=64, 47.8%; Embase: n=26, 19.4; and IEEE Xplore: n=19, 14.2%). After removing duplicates (57/134, 42.5%), we screened the remaining articles (77/134, 57.5%). Of these 77 articles, 69 (90%) met the inclusion criteria, and 8 (10%) were excluded. After full-text analysis, of the 69 articles, 50 (72%) were excluded, and 19 (28%) studies involving 5859 participants were included in the review synthesis (Figure 1). The included studies were published between 2016 and 2025.

### Quality of the Studies and Risk-of-Bias Assessment

**Figure 2:**
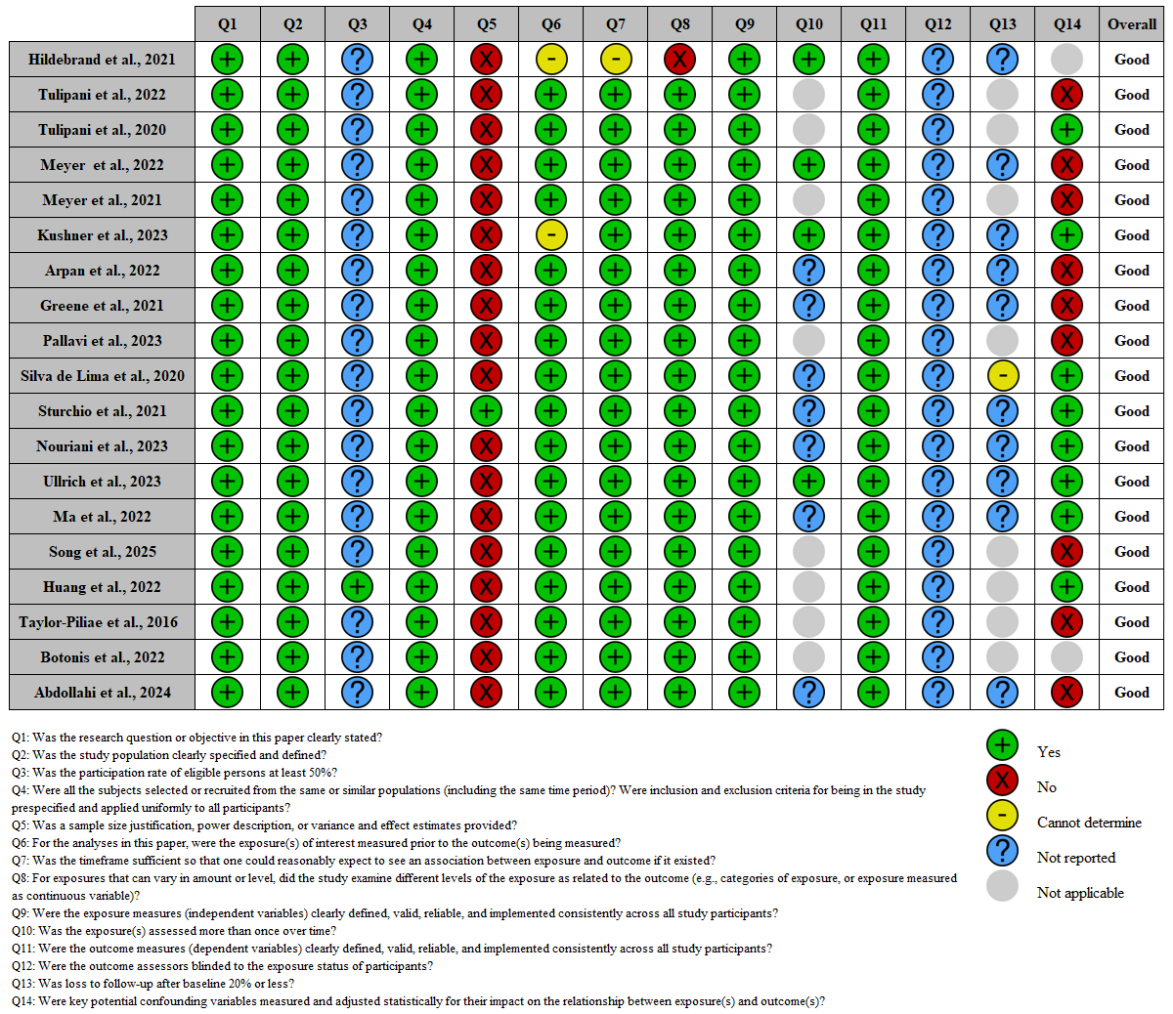
Risk-of-bias assessment for included studies [23,35-52].

The risk of bias in the included studies was assessed using the National Heart, Lung, and Blood Institute Quality Assessment Tool for Observational Cohort and Cross-Sectional Studies, which comprises 14 questions (Figure 2) 

All included studies were rated by the reviewers as having overall good quality (Figure 2), and there was homogeneity in the methodologies and procedures used to assess fall risk in neurological disorders. However, concerns were identified in some domains of the quality assessment tool, specifically items 3, 5, 12, and 13; for example, participation rates of eligible individuals were documented in only 5% (1/19) of the studies [50], a sample-size justification or power analysis was provided only in the study by Sturchio et al [39], repeat assessment of the exposure was described in only 21% (4/19) of the studies [48,49,51,52], and 79% (15/19) of the studies relied on a single measurement or used a cross-sectional design. Moreover, procedures for blinding outcome assessors to exposure status were not reported, and information sufficient to determine loss to follow-up was not provided in the prospective cohort studies. However, these reporting limitations did not affect the overall quality classification assigned to each study.

### Study Population

The included studies involved 2630 patients with neurological disorders, including 226 (8.59%) with MS (n=7, 37% studies), 2305 (87.64%) with PD (n=10, 53% studies), 51 (1.94%) with stroke (n=3, 16% studies), and 48 (1.83%) with AD or cognitive impairment (n=1, 5% study). An additional 3229 participants were healthy controls or older adults. Regarding disease severity, the studies conducted in patients with MS had a mean Expanded Disability Status Scale score of 3.4; and in patients with PD, the mean score on the Unified Parkinson’s Disease Rating Scale, part III (motor examination), was 26.6, while the mean Hoehn and Yahr stage was 2.58 (based on the studies that reported these metrics). Regarding patients with stroke, 2 (11%) of the 19 studies [36,38] included participants who had experienced a cerebrovascular accident at least 5 years earlier, while 1 (5%) included patients who had experienced a stroke at least 6 months before the day of the experiment [40] (Table 2).

**Table 2:** Descriptive summary of the selected studies.

Neurological disorders and studies		Study design	Sample size, n (etiology)	Demographics	Study aim	Major findings
**MS^a^**						
	Hildebrand et al [51], 2021	Prospective cohort study	25 (MS)	Female: 17; male: 8; EDSS^b^: median 6.0 (IQR 5.5-6.0); RR^c^: 11; SP^d^: 8; PP^e^: 6	The study compared the sensitivity and false discovery rates of 3 fall detection methods: prospective paper fall calendars, real-time self-reporting, and automated detection using a body-worn device	Fall calendars likely underestimate fall frequency by approximately 40%, whereas the automated detector misses very few falls but likely overestimates the number of falls by approximately 1 per day
	Tulipani et al [23], 2022	Cross-sectional methodological case-control study	37 (MS—nonfallers: 17; fallers: 21)	EDSS—nonfallers: mean 2.0 (SD 0.8); fallers: mean 3.3 (SD 1.4)	Participants completed the 30CST^f^ in both supervised (laboratory) and unsupervised (home) settings while wearing a thigh-mounted triaxial accelerometer; in the unsupervised setting, they also performed 30CSTs every 2 h and rated their balance confidence and fatigue over 48 h	Short periods of instrumented, unsupervised monitoring, in addition to regular clinical assessments, could enhance the accuracy of fall risk prediction
	Tulipani et al [41], 2020	Cross-sectional methodological case-control study	38 (MS—nonfallers: 17; fallers: 21)	EDSS—nonfallers: mean 2.3 (SD 1.2); fallers: mean 3.4 (SD 1.2)	Participants completed self-report measures and performed the 30CST, with triaxial acceleration data collected from sensors on the thigh and chest; the study assessed the relationship between accelerometer metrics and clinical measures and developed a logistic regression model to classify fall status	Accelerometer-derived metrics were linked to clinically relevant measures of disease severity, fatigue, and balance confidence during balance-challenging tasks, suggesting that inertial sensors could improve functional assessments and fall risk identification, with their simplicity aiding community-based monitoring
	Meyer et al [48], 2022	Cross-sectional study with follow-up	38 (MS—nonfallers: 17; fallers: 21)	Male: 12; female: 26; EDSS—nonfallers: mean 2.3 (SD 1.0); fallers: mean 3.3 (SD 1.4)	Participants were categorized as fallers or nonfallers based on self-reported falls, underwent clinical assessments, completed balance and fatigue surveys, performed laboratory-based mobility tests while wearing MC10 BioStamp sensors to collect accelerometer and EMG^g^ data, and continued to wear the sensors at home for 48 h to monitor acceleration	Deep learning models performed better than feature-based models when analyzing home data, particularly when using all walking bouts for deep learning and shorter bouts for feature-based models in individual bout evaluations; longer free-living walking bouts showed more pronounced differences between fallers and nonfallers compared to shorter bouts, and aggregating data from all free-living walking bouts resulted in the highest accuracy for fall risk classification
	Meyer et al [37], 2021	Cross-sectional methodological case-control study	37 (MS—nonfallers: 19; fallers: 18)	Male: 11; female: 26; EDSS—mean 2.99 (SD 1.47)	The study distinguished fallers from nonfallers based on a 1-min walking task; participants were evaluated using wearable sensors to collect accelerometer data	The BiLSTM^h^ deep learning model achieved the highest performance with an AUC^i^ of 0.88, suggesting that deep learning using gait acceleration data is effective for classifying fall status
	Kushner et al [52], 2023	Prospective cohort study	25 (MS)	Male: 8; female: 17; EDSS: median 6.0 (IQR 5.5-6.0); RR: 11; SP: 8; PP: 6	Participants were monitored over 8 wk using an inertial sensor equipped with a triaxial accelerometer and time-of-flight radio transmitter, communicating with beacons placed throughout the home, to assess associations between home locations and movement behaviors before falls compared to periods without falls	Movement metrics obtained from wearable sensors and smart-home tracking systems were correlated with fall risk, with increased pauses during walking and more complex, longer movement paths indicating higher fall risk
	Arpan et al [44], 2022	Prospective cohort study	26 (MS—nonfallers: 13; fallers: 13)	EDSS—nonfallers: mean 4.3 (SD 0.23); fallers: mean 4.2 (SD 0.18)	The study computed the AUC values for each gait measure that differentiated fallers from nonfallers and then ranked values from highest to lowest to assess their effectiveness in predicting falls based on instrumented measures of mobility using a univariate model	Objective monitoring of gait and turning in daily life identified those at risk of future falls, with the pitch at toe-off identified as the most significant predictor, suggesting potential benefits from interventions targeting plantarflexion muscle strength, range of motion, and proprioceptive input
**PD^j^**						
	Greene et al [46], 2021	Cross-sectional study with follow-up	1057 (PD [more impaired]: 15; PD [less impaired]: 26; controls: 1015)	Participants with PD (more impaired)—male: 10; female: 5; participants with PD (less impaired)—male: 17; female: 9; UPDRS-III^k^—participants with PD (more impaired): mean 15.1 (SD 9.6); participants with PD (less impaired): mean 22.56 (SD 10.25); healthy controls—male: 344; female: 671	The study analyzed data using 2 statistical approaches to predict fall counts: a previously reported fall risk assessment algorithm as well as elastic net and ensemble regression models	The study found a robust correlation between fall counts and a previously established inertial sensor–based fall risk estimate, as well as significant associations between fall counts and various individual gait and mobility parameters, suggesting that falls predicted from inertial sensor data during a basic walking task could serve as a promising digital biomarker for PD warranting further validation in clinical settings
	Pallavi et al [35], 2023	Cross-sectional study	28 (PD: 14; healthy controls: 14)	Participants with PD—male: 11; female: 3; UPDRS: mean 35.5 (SD 19.4); H&Y^l^: mean 2.2 (SD 0.7); healthy controls—male: 11; female: 3	Participants were asked to walk overground on a 10-m pathway, both without turns and with 180° turns, under varying task conditions of increasing complexity (single-, dual-, and multiple-task conditions)	The knee flexion and gait-related indices strongly aligned with clinical measures of fear of falling, especially in the group with PD, providing valuable predictive information for clinicians
	Silva de Lima et al [42], 2020	Prospective cohort study	4126 (PD: 2063; healthy controls: 2063)	Participants with PD—male: 996; female: 1067; healthy controls—male: 992; female: 1071	The study used a PERS^m^, which consisted of a device—worn as a necklace—with multiple embedded sensors for automatic fall detection and manual fall reporting; fall events were reported either by a button push or automatically detected by the fall detector	Wearable sensors were used to collect fall data, revealing that PD nearly doubles fall incidence in everyday life, underscoring PD as a significant fall risk; in addition, monitoring fall events in >4000 participants using a wearable sensor connected to a PERS suggests the potential of body-worn sensors for continuous home monitoring
	Sturchio et al [39], 2021	Prospective cohort study	26 (PD and OH^n^)	Male: 19; female: 7; UPDRS-III: mean 40.1 (SD 13.4)	The study estimated the association of PD and OH with fall risk association with fall risk, capturing fall frequency through a diary over 6 mo	Kinematic measures, rather than clinical assessments, predicted falls in PD with OH, suggesting that orthostatic mean arterial pressure of ≤75 mm Hg may indicate a hemodynamic threshold associated with increased fall risk, advocating for proactive corrective interventions
	Nouriani et al [43], 2023	Cross-sectional algorithm validation with prospective cohort	21 (PD: 11; healthy controls: 10)	UPDRS pull test: mean 0.56 (SD 0.96)	The study validated the activity recognition algorithm through video footage of participants with PD wearing sensors at home	The findings suggest that incorporating near-fall detection into home monitoring data significantly enhances the performance of fall prediction algorithm
	Ullrich et al [49], 2023	Prospective cohort study	35 (PD—nonfallers: 25; fallers: 10)	Male: 26; female: 9; UPDRS—nonfallers: mean 12.9 (SD 6.2); fallers: mean 21.7 (SD 9.1); H&Y—nonfallers: mean 2.3 (SD 0.5); fallers: mean 2.9 (SD 0.5)	The study compared various data aggregation methods and machine learning models for predicting fall risk using gait parameters obtained from continuous real-world recordings and unsupervised gait tests	The findings indicate that combining 2 wk of real-world gait data provides the most accurate prediction of fall risk, surpassing predictions based solely on unsupervised gait tests (balanced accuracy of 68%), thereby advancing understanding in fall risk prediction
	Ma et al [45], 2022	Prospective cohort study	51 (PD)	Male: 33; female: 18; UPDRS-III: mean 33.6 (SD 13.5); H&Y: mean 2.4 (SD 0.8)	Using wearable sensors, the study objectively evaluated gait characteristics in participants with PD who experienced falls; it also explored the relationship between spatiotemporal gait parameters, gait variability, and falls over a 6-mo follow-up period	Increased gait variability is a notable characteristic among individuals with PD who experience falls and proves more sensitive in identifying those at elevated risk of falls than spatiotemporal parameters
	Song et al [47], 2025	Cross-sectional case-control study	111 (PD: 29; healthy controls: 18 [dataset III]; PD: 35; healthy controls: 29 [dataset IV])	Participants with PD—male: 20; female: 9; age: mean 71.1 (SD 8.1) y; healthy controls—male: 10; female 8; age: mean 71.6 (SD 6.7) y (dataset III); participants with PD—male: 22; female: 13; age: mean 61.6 (SD 8.9) y; healthy controls—male: 18; female 11; age: mean 57.9 (SD 7.0) y (dataset IV)	The study identified an abnormal gait pattern termed recessive weak foot, characterized by a discontinuous high-risk gait on the weak foot side, observed through weak foot feature space; to address this, the authors proposed a trainable threshold method to discriminate individuals with this pattern, thereby enhancing the model’s generalization performance; the authors conducted feasibility and ablation studies on 2 self-established datasets and tested the compatibility on 2 published gait-related datasets of participants with PD	Guided by a customized index and the optimized adaptive thresholds, the method used by the authors effectively screened out individuals with recessive weak foot; specifically, after fine adaptation, the individual-specific models could achieve accuracies of 87.5% and 73.6% on an enhanced dataset; compared to the baseline, the proposed 2-stage model demonstrated improved performance, with an accuracy of 85.4% and sensitivity of 87.5%; in the dataset of participants with PD, the authors’ method mitigated potential overfitting from low feature dimensions, increasing accuracy by 4.7%
**AD^o^**						
	Huang et al [50], 2022	Cross-sectional study	103 (older adults with CI^p^, including those with aMCI^q^ and mild AD: 48; cognitively healthy older adults: 55)	Participants with CI—male: 21; female: 27; age: mean 65.7 (SD 5.2) y; cognitively healthy participants—male: 15; female: 40; age: mean 67.1 (SD 4.1) y	The study primarily examined the association between memory deficit and increased fall risk; it also explored the underlying neuroanatomical linkage of this association in older adults with aMCI and mild AD	The authors’ findings suggested that memory deficit was associated with increased fall risk in older adults with aMCI and mild AD; the association might be mediated by the atrophy of the medial temporal, frontal, and parietal lobes; in addition, increased fall risk, tested by timed up and go time, heel stride angles, and stride speed, might be objective and convenient kinematics markers for dynamic monitoring of both memory function and fall risk
**Stroke**						
	Taylor-Piliae et al [36], 2016	Cross-sectional feasibility study	20 (stroke: 10 [ischemic stroke: 6; hemorrhagic stroke: 4]; healthy controls: 10)	Participants with stroke: female: 7; male: 3; healthy controls: female: 9; male: 1; poststroke duration: mean 42 (SD 25) mo	The study assessed the feasibility of using a kinematic motion sensor (PAMSys) to monitor fall risk and gait in community-dwelling survivors of stroke by evaluating the acceptability of wearing the sensor for 48 h, identifying fall risk indicators and gait parameters, and comparing these metrics with data from age-matched nonfrail controls	Survivors of stroke found 48 h of continuous PAMSys monitoring highly acceptable, suggesting that in-home wearable technology could be valuable for monitoring fall risk and gait, potentially aiding in recovery efforts
	Botonis et al [38], 2022	Cross-sectional study	35 (stroke: 20; healthy controls: 15)	Participants with stroke—male: 10; female: 10; poststroke duration: mean 7.47 (SD 4.58) y; healthy controls—male: 8; female: 7	The study investigated whether population-specific training data and modeling parameters were necessary to predetect falls in individuals with chronic stroke	The findings underscore the significance of population-specific sensitivity, use of nonfall data, and optimal lead time for machine learning–based preimpact fall detection tailored for individuals with stroke, suggesting a need for the inclusion of individuals with neurological impairments in model development for effective fall detection in other populations considered to be at high risk
	Abdollahi et al [40], 2024	Prospective cohort study	21 (stroke)	Male: 11; female: 10; age: mean 66 (SD 10) y	The study aimed to develop machine learning models using inertial sensors to objectively classify fall risk in survivors of stroke	The random forest model achieved 91% accuracy using dual-task balance sway and timed up and go walk time features; single thorax sensor models performed similarly to multisensor models; balance and timed up and go best predicted fall risk

^a^MS: multiple sclerosis.

^b^EDSS: Expanded Disability Status Scale.

^c^RR: relapsing-remitting.

^d^SP: secondary progressive.

^e^PP: primary progressive.

^f^30CST: 30-second chair stand test.

^g^EMG: electromyography.

^h^BiLSTM: bidirectional long short-term memory.

^i^AUC: area under the receiver operating characteristic curve.

^j^PD: Parkinson disease.

^k^UPDRS-III: Unified Parkinson’s Disease Rating Scale, part III (motor examination).

^l^H&Y: Hoehn and Yahr.

^m^PERS: personal emergency response system.

^n^OH: orthostatic hypotension.

^o^AD: Alzheimer disease.

^p^CI: cognitive impairment.

^q^aMCI: amnestic mild cognitive impairment.

### Classification of the Results

We classified our results according to the type of motor task (supervised vs unsupervised), the biomechanical parameters extracted, and the technological equipment used as well as the artificial intelligence (AI) approach applied in the included studies.

#### Motor Task for Fall Risk Detection

Of the 19 included studies, 12 (63%) assessed fall risk through supervised motor tasks, administered in clinical or laboratory settings, while the remaining 7 (37%) evaluated fall risk through unsupervised gait tasks recorded in real-world environments.

#### Supervised Motor Tasks

Generally, supervised motor tasks consisted of instrumented versions of the TUG test [39,41,45,46,48], the 30-second chair stand test (30CST) [23,41,48], and simulated daily activities [37,39]. However, Tulipani et al [23] compared supervised 30CST with unsupervised 30CST in a home-based setting. The authors reported a substantial statistical difference between the 2 types of motor tasks, suggesting that the laboratory setting could influence the fall risk rate. Some of the studies (5/19, 26%) [37,39,41,45,48] administered a specific assessment protocol to quantify the risk of falls in patients with neurological disorders, while the studies by Pallavi et al [35] and Greene et al [46] quantified fall risk using solely TUG or gait tasks. Similarly, the study by Song et al [47] analyzed different datasets in which the only motor task consisted of walking along a 20-meter corridor for at least 2 minutes under observation. Interestingly, Sturchio et al [39] conducted an instrumented assessment of activities of daily living (ADLs) in a standardized home-like environment, in addition to clinical tests (eg, the TUG test, the 2-minute walk test, and other standard measures; Table 2). The TUG test was also used to assess gait function in patients with AD [50]. Similarly, Meyer et al [37], acquired sensor data from simulated daily activities in addition to several standard functional assessments. In the study by Abdollahi et al [40], patients with stroke completed clinical tasks, including balance tests (eyes open and closed), the TUG test, the 10-meter walk test, and the sit-to-stand test. Each was also performed as a motor-cognitive dual task, involving backward counting during movement. Finally, Botonis et al [38] evaluated fall risk in patients with stroke through balance response reactivity in a laboratory setting.

#### Unsupervised Motor Tasks

Of the 19 included studies, 7 (37%) [36,42-44,49,51,52] assessed fall risk using unsupervised recordings in a real-world environment. In general, these assessments were conducted during ADLs [36,43,51] or gait tasks [44,49,52]. Notably, Silva de Lima et al [42] detected fall events automatically using a wearable sensor, without monitoring ADLs or gait.

#### Fall Risk Biomechanical Parameters

We analyzed features extracted from wearable sensors related to the quantification of supervised or unsupervised motor tasks in patients with MS, PD, or stroke. Several of the studies (10/19, 53%) [35,36,39-41,45,47-49,52] extracted specific gait parameters from wearable sensors. These parameters consisted mainly of spatiotemporal parameters of gait [35,45,49], anterior-posterior and medial-lateral acceleration [41], lower limb joint angles, and cadence [35]. In particular, Arpan et al [44] extracted specific gait parameters to differentiate fallers from nonfallers. These biomechanical parameters reflected gait quality (eg, gait speed, stride length, cadence, and swing) as well as turning function [44]. In addition, Song et al [47] extracted 44 gait features mainly from plantar pressure data, focusing on the weaker foot. These features were used to identify a novel gait dysfunction, termed recessive weak foot, which was characterized by discontinuous high-risk gait patterns. Furthermore, Abdollahi et al [40] and Huang et al [50] extracted spatiotemporal gait parameters derived from the clinical tests (the TUG test and the 10-meter walk test). Only 2 (11%) of the 19 studies [36,39] extracted balance features in addition to gait parameters. In particular, Sturchio et al [39] evaluated oscillations in the center of pressure and center of mass, the frequency of oscillations, and jerkiness in patients with PD. The authors also evaluated upper limb range of motion during motor tasks. Similarly, Arpan et al [44] evaluated upper body kinematics alongside gait quality through spatiotemporal parameters and additionally calculated specific parameters during gait turning. Gait turning and postural transfers were also analyzed by Greene et al [46], while Tulipani et al [23,41] focused on specific biomechanical parameters during the 30CST (Table 3).

As shown in Table 3, the majority of the included studies (18/19, 95%) analyzed gait parameters, such as spatiotemporal features. These features were mostly considered in patients with PD or MS. In addition, some of the studies (7/19, 37%) extracted biomechanical features from clinical tests, such as 30CST and TUG (eg, TUG time, turning activity, 30CST average time, and repetitions). Concerning patients with stroke, it was difficult to define a common pattern of extracted biomechanical features because only 3 studies were available; however, both considered fall characteristics and parameters related to postural changes, similar to those examined in studies involving patients with PD or MS [43,52].

**Table 3:** Biomechanical features—analyzed to quantify fall risk in patients with multiple sclerosis, Parkinson disease, Alzheimer disease, or stroke—extracted from wearable sensors in the included studies.

Neurological disorders and studies		Motor task	Parameters analyzed
**Multiple sclerosis**			
	Hildebrand et al [51], 2021	Unsupervised tasks during daily life activities	Acceleration signals from triaxial accelerometers mounted on the waist TUGa measurements for assessing balance and mobility in participants Location tracking using GPS and time-of-flight sensors to associate falls with specific movements or environments
	Tulipani et al [23], 2022	30CST^b^—the participant was encouraged to complete as many full stands as possible within 30 s	Metrics for sit-to-stand and stand-to-sit transitions: Sit-to-stand time (average, maximum, and minimum time across 30CST repetitions) Stand-to-sit time (average, maximum, and minimum time across 30CST repetitions) Total number of 30CST repetitions
	Tulipani et al [41], 2020	Participants performed a series of functional assessments, including 1 trial each of the 30CST, T25W^c^, and TUG, as well as a 30-s static standing trial with instructions to maintain a tall posture with their feet facing forward, wearing inertial sensors on the anterior right thigh and chest	Metrics for sit-to-stand and stand-to-sit transitions: Mean and coefficient of variation of sit-stand time and stand-sit time Peak cranial-caudal, anterior-posterior, and medial-lateral acceleration during transitions
	Meyer et al [48], 2022	Supervised tasks completed in the following order: TUG; T25W; 30CST; lying-to-standing transition; 3 separate 2-minute standing tests (tandem standing, feet shoulder width apart with eyes open, and feet shoulder width apart with eyes closed); 1-min hallway walk at a self-selected pace, including 1 turn; 30-s normal standing; 30-s upright sitting; 30-s slouch sitting; and 30 s each lying on the back, left side, right side, and prone	Gait parameters extracted: Stance time, swing time, stride time, and variability measures (coefficient of variation of stride and duty factor) Nonlinear measures (entropy ratio and Lyapunov exponent) Root mean square of the anterior-posterior acceleration Medial-lateral frequency dispersion
	Meyer et al [37], 2021	Data from these sensors were recorded during a variety of simulated daily activities and several standard functional assessments	Spatiotemporal parameters of gait stride
	Kushner et al [52], 2023	Unsupervised tasks during daily life activities (eg, gait quality and turning)	Gait initiation time Spatiotemporal parameters of gait (eg, average step length, average walking speed, and percentage of time spent turning while walking) Time spent moving Movement length An entropy-based metric quantifying movement complexity through transitions between rooms Mean walking path length inside the home
	Arpan et al [44], 2022	Unsupervised tasks during daily life activities (eg, gait quality and turning)	Instrumented measures of mobility as predictors of future falls: Pitch at toe-off Gait speed Stride length Double support Swing Pitch at initial contact Turn angle
**Parkinson disease**			
	Greene et al [46], 2021	Supervised TUG test, instrumented with inertial sensors	71 different calculated parameters quantifying gait, mobility, turning, and transfers, along with a statistical fall risk estimate and frailty estimate based on inertial sensor data: TUG time Gait velocity Fall risk estimate Frailty estimate Mobility impairment scores
	Pallavi et al [35], 2023	Gait tasks: single, dual, and multiple	Gait parameters: Knee flexion during heel-strike event Knee flexion during toe-off event Cadence Double limb support time
	Silva de Lima et al [42], 2020	Unsupervised; fall events were collected either automatically using the wearable fall detector or were registered by a button push on the same device	Fall event (manually detected) Fall event (automatically detected) Changes in height Changes in orientation Impact during fall
	Sturchio et al [39], 2021	Participants performed the following tasks: lying-to-standing test (standing up without assistance after 10 min of supine resting and keeping the upright position for 5 min), tests of gait and postural stability (TUG, 2MWT^d^, and sway [eyes opened and eyes closed]), and ADLs^e^ conducted in a standardized home-like environment	Balance parameters: Oscillations in the CoPf Oscillations in the CoMg Jerk sway (jerkiness) Root mean square sway (magnitude of acceleration) CFh sway (frequency of oscillation) Gait parameters: Turn duration Total duration Peak turn velocity Waist sway during TUG Gait speed Stride length Number of steps during turning Range of motion of upper limbs Cadence
	Nouriani et al [43], 2023	Unsupervised tasks during daily life activities	IMUi values: Time spent lying down per day (lie-down frequency, ie, lying duration/total time) Total number of ambulatory bouts at home Frequency of sitting at home (eg, sitting duration/total time) Walking frequency at home Peak acceleration of the chest at home
	Ullrich et al [49], 2023	Unconstrained real-world gait analysis and unsupervised gait tasks	Spatiotemporal gait parameters, including the following: Stride time Stance time Swing time Stride length Gait speed Initial contact foot angle Final contact foot angle Maximum foot lift
	Ma et al [45], 2022	Participants were required to complete a 7-meter TUG test twice (stand up from an armless chair, walk at own comfortable pace for 7 meters, turn 180°, then walk back and sit down)	Spatiotemporal gait parameters, including the following: Stride length Gait cycle time Gait phase (swing and double support percentages) Range of motion of the trunk in the sagittal plane Gait variability measures
	Song et al [47], 2025	Participants were asked to walk for at least 2 min consecutively at their normal gait and speed along a 20-meter-long corridor	44 CoP features based on recessive weak foot were extracted: Weak and single foot features: mean and SD of medial-lateral anterior-posterior CoP, mean and SD of resultant distance, total excursions, and confidence circle area Symmetry-based features: Gait asymmetry, similarity, and divergence Temporal consistency–based features: Gait inconsistency, sequential similarity, and sequential Jensen-Shannon divergence
**Alzheimer disease**			
	Huang et al [50], 2022	Supervised TUG test	TUG test time Heel-strike angles Stride speed Cadence
**Stroke**			
	Taylor-Piliae et al [36], 2016	Unsupervised tasks during daily life activities	Parameters related to postural transitions (eg, sit-to-stand, standing-to-sitting, lying-to-sitting, and sitting-to-lying tasks): Duration (s) Number of unsuccessful attempts Gait parameters (detected based on the peaks in the vertical accelerometer data, following predefined conditions): Number of steps taken Speed of walking (meters per s) Duration of walking activities (percentage of total activity)
	Botonis et al [38], 2022	Participants were instructed to respond to loss of balance using any natural technique, such as using an arm to catch themselves or taking multiple steps, to encourage realistic behavior	Preimpact data: Fall impact and acceptable intervention time Statistical features from raw IMU signals, including the following: Minimum Median Maximum IQR SD Skew Kurtosis
	Abdollahi et al [40], 2024	Balance test (stand as still as possible for 30 s with eyes open or closed on a firm surface), TUG, 10MWT^j^, STS^k^, and motor-cognitive dual tasks (repetition of the aforementioned tasks while counting backward from 200 by 10s)	Biomechanical parameters extracted during clinical tests: Walk time Step cadence Mean and SD of swing total, single support, and stride duration Thorax, thigh, and pelvis linear acceleration Thorax and pelvis angle velocity TUG and STS time TUG: walk toward cone, turn around the cone, walk toward chair, turn and sit, steps toward cone, steps toward chair, cadence toward cone, and cadence toward chair

^a^TUG: timed up and go.

^b^30CST: 30-second chair stand test.

^c^T25W: timed 25-foot walk.

^d^2MWT: 2-minute walk test.

^e^ADL: activity of daily living.

^f^CoP: center of pressure.

^g^CoM: center of mass.

^h^CF: centroidal frequency.

^i^IMU: inertial measurement unit.

^j^10MWT: 10-meter walk test.

^k^STS: sit-to-stand.

#### Technological Equipment, Data Processing, and Analysis Approaches

This subsection describes the technological equipment and data processing methods used across the included studies. Of the 19 studies, 7 (37%) [23,37,41,44,48,51,52] focused on fall risk assessment in patients with MS using wearable sensors. Of these 7 studies, 4 (57%) used the MC10 BioStamp system, which integrates triaxial accelerometers, gyroscopes, and electromyography (EMG) sensors to capture a wide range of motion data [23,37,41,48]. Tulipani et al [41] examined accelerometer metrics from thigh and chest sensors and applied logistic regression models to distinguish fallers from nonfallers, achieving an area under the receiver operating characteristic curve (AUC) of 0.78 and an accuracy of 74%. In the study by Tulipani et al [23], a single sensor placed on the thigh was used to record data during tests, and the authors used receiver operating characteristic analysis to optimize classification thresholds. This improved predictive performance in unsupervised settings, with an AUC of 0.79 and an accuracy of 78.4%.

Meyer et al [37] used BioStamp in conjunction with APDM Opal wearable sensors. Data were acquired from triaxial accelerometer sensors secured to the sternum (below the clavicle) and to the anterior portion of the right thigh. In addition, the authors recorded data from accelerometers and angular rate gyroscopes secured to the lower sternum, lower back (at the belt line), and anterior right and left shanks. The authors demonstrated that a bidirectional long short-term memory (LSTM) deep neural network could identify patients with MS who had recently fallen, achieving an AUC of 0.88 and an accuracy of 86%, based on data collected from 2 wearable sensors during a 1-minute task [37].

In the study by Meyer et al [48], accelerometer and EMG data were collected from the right and left tibialis anterior, while accelerometer and angular rate gyroscope data were collected from the chest and lower back as well as bilaterally from the anterior thighs, proximal lateral shank, and dorsal aspect of the feet. EMG data were collected to allow the investigation of foot drop. The models tested in this study showed that short walking bouts were the best predictor of fall risk in free-living conditions (AUC=0.63). By contrast, medium and long walking bouts had lower predictive performance, with AUCs of 0.52 and 0.54, respectively. DL models, especially LSTM models that used up to 22 strides, outperformed traditional feature-based models, with the LSTM model achieving an AUC of 0.76, showing its robustness in discerning fall risk from complex gait patterns. Hildebrand et al [51] and Kushner et al [52] used an automated fall detection system that integrated a body-worn IMU (placed on the waist) with a button for self-reporting falls and an ML algorithm trained to evaluate falls in real-world environments in patients with MS [53]. Specifically, the algorithm used an autoencoder that identifies individuals at increased risk of falling using reconstruction errors in accelerometer signals, followed by a hyper-ensemble of balanced random forests trained on acceleration and movement features. Hildebrand et al [51] compared 3 methods for detecting falls in patients with MS: traditional paper fall calendars, real-time self-reporting using a body-worn device, and automated fall detection using the same device. The results showed that paper calendars had a sensitivity of 61.4% and a false discovery rate of 6.7%, indicating that they likely underestimated the number of falls. By contrast, the automated detection system had a high sensitivity of 92.1% but also a high false discovery rate of 91.9%, indicating that it likely overestimated fall occurrences. The study highlighted the challenges of balancing high sensitivity with minimizing false positives in real-world applications. Kushner et al [52] focused on extracting movement complexity metrics from wearable sensors. The study used a k-nearest neighbors ML algorithm for room detection and applied complex statistical methods to estimate movement-based fall risk. The findings indicated that movement complexity, measured by the entropy of room transitions, was notably higher in the periods leading up to falls, suggesting that increased movement complexity is a strong predictor of fall risk. Arpan et al [44] used a different set of wearable technologies to passively monitor gait and turning behaviors in daily life to predict falls in patients with MS. This setup involved triaxial accelerometers, gyroscopes, and magnetometers (Opal sensors) placed on the top of the foot and integrated into instrumented socks, along with a sensor worn on the lower lumbar region. The data collected from these sensors were processed using an unscented Kalman filter technique to fuse accelerometer and gyroscope data and identify walking bouts and turning behaviors. The raw data were further processed using commercial gait analysis algorithms in Mobility Lab to derive spatiotemporal measures of gait and turning. The study found that reduced plantarflexion during toe-off, reflected by the pitch angle of the foot during the push-off phase of walking, was the most significant predictor of falls, achieving an AUC of 0.86, indicating strong predictive power [44].

Of the 19 studies, 8 (42%) [35,39,42,43,45-47,49] focused on integrating advanced wearable sensors and complex data analysis methods to monitor and predict fall risks in patients with PD. Silva de Lima et al [42] used a wearable sensor worn as a necklace (Philips Lifeline FD100) to monitor falls in patients with PD in their home environments. Although the study did not predict falls, it revealed a significantly higher incidence of falls in patients with PD compared to controls, demonstrating the pronounced fall risk in this population [42]. Song et al [47] analyzed PD gait data from intelligent footwear with 16 plantar pressure sensors embedded in each insole. The authors proposed a 2-stage model that first identifies individuals with PD with irregular gait patterns, referred to as recessive weak foot, using an adaptive threshold based on the distribution difference index. In the second stage, separate personalized ML models (such as support vector machines, logistic regression, and decision trees) are trained for individuals with recessive and dominant weak foot patterns, enabling a more accurate and individualized assessment of fall risk.

Of the 19 studies, 3 (16%) used compact gait analysis systems to collect comprehensive kinematic data. Ma et al [45] and Sturchio et al [39] used the Mobility Lab system, which includes 6 wearable sensors (accelerometers, gyroscopes, and magnetometers) placed on the feet, wrists, sternum, and lumbar region. Ullrich et al [49] used the Mobile GaitLab system, with sensors positioned on the instep of both the left and right shoes. Similarly, Huang et al [50] used the JiBuEn gait analysis system, which consists of wearable sensors embedded in specially designed shoes. Ma et al [45] focused on gait variability as a predictive marker for falls in patients with PD, capturing metrics such as stride length, gait cycle time, and range of motion of the trunk in the sagittal plane. The authors found that variability in the trunk range of motion during walking was a significant independent risk factor for falls in patients with PD, achieving a prediction accuracy with an AUC of 0.75. The overall logistic regression model, which included multiple gait parameters, predicted falls with a higher accuracy with an AUC of 0.84 [45]. Sturchio et al [39] focused on fall risk in patients with PD with orthostatic hypotension and found that waist sway, jerkiness, and postural sway were more predictive of falls than traditional clinical assessments, achieving an AUC of ≥0.81 for predicting fall risk. Ullrich et al [49] focused on fall risk prediction in patients with PD by analyzing real-world gait data. The study used ML algorithms, notably random forest classifiers, to analyze the aggregated gait data for fall risk prediction. The highest predictive performance was achieved using participant-wise aggregated data, resulting in a balanced accuracy of 74%, with a sensitivity of 60% and a specificity of 88% [49].

Greene et al [46] explored the utility of wearable sensors during the TUG test to develop a digital biomarker for predicting fall counts. The study applied 2 statistical approaches to predict fall counts: a fall risk assessment algorithm reported in previous studies by the same team [54,55] and an ensemble model combining elastic net and regression models using Poisson regression. The research findings suggest a strong association between the fall counts predicted from the inertial sensor data and actual falls [46]. Nouriani et al [43] validated an activity recognition algorithm and developed novel behavioral biomarkers to assess fall risk in patients with movement disorders, focusing on PD and normal pressure hydrocephalus. The study used 5 IMUs strategically placed on the chest, upper legs, and lower legs of participants, capable of measuring acceleration, angular rates, and orientation. The core of the study’s methodology involved a DL-based activity recognition architecture using a convolutional neural network combined with LSTM cells. This advanced ML approach allowed for the accurate detection of complex activities and postural transitions that are critical indicators of fall risk. The algorithm’s performance was validated against video [43], achieving high sensitivity (>95%) in detecting activities and an 80% sensitivity for near falls.

Finally, Pallavi et al [35] used a novel wearable sensor (SmartWalk) to evaluate knee flexion and gait indices during walking tasks under different cognitive load conditions. The study found that variability in knee flexion increased significantly under dual-task conditions, which were closely associated with a fear of falling. The findings indicate significant increases in the variability of knee flexion during walking tasks, particularly under dual- and multiple-task conditions, which pose higher cognitive demands and thus exacerbate gait and postural disturbances in patients with PD. A strong relationship between measured indices and clinical assessments of fear of falling was found using statistical analyses, including k-means clustering and correlation assessments [35].

Of the 19 studies, 3 (16%) [36,38,40] focused on fall assessment in survivors of stroke. Botonis et al [38] investigated the efficacy of wearable airbag technology paired with ML models to prevent falls. The device included 3 IMU sensors (on the hips and lower back) and used adaptive boosting classifiers to differentiate fallers from nonfallers. Two models were developed: one trained on survivors of stroke and the other on control data. The stroke-specific model significantly outperformed the control model in detecting true falls, especially in classifying complex fall movements in anterior-posterior directions. In addition, training the model with daily activity data enhanced its accuracy in fall classification [38]. Taylor-Piliae et al [36] explored the feasibility of using a wearable sensor technology (PAMSys) for objective fall risk and gait monitoring in community-dwelling survivors of stroke. The triaxial accelerometer was worn in a midsternal pocket of a custom T-shirt, continuously recording movement data focused on postural transitions, such as the sit-to-stand test. Data on steps, speed, and movement transitions were recorded over 48 hours and compared with controls. The analysis showed that survivors of stroke exhibited worse fall risk indicators, taking longer to change posture and having more failed sit-to-stand attempts than controls [36]. In another study [40], 8 IMUs (placed on feet, shanks, thighs, pelvis, and thorax) per participant were used. The authors analyzed the gait kinematic data extracted from IMUs with ML algorithms. In particular, they used classification algorithms, including random forest, support vector machine, and logistic regression. Among these, the random forest achieved 91% accuracy using just 2 features: thorax mediolateral acceleration during dual-task balance and TUG walk time.

**Figure 3:**
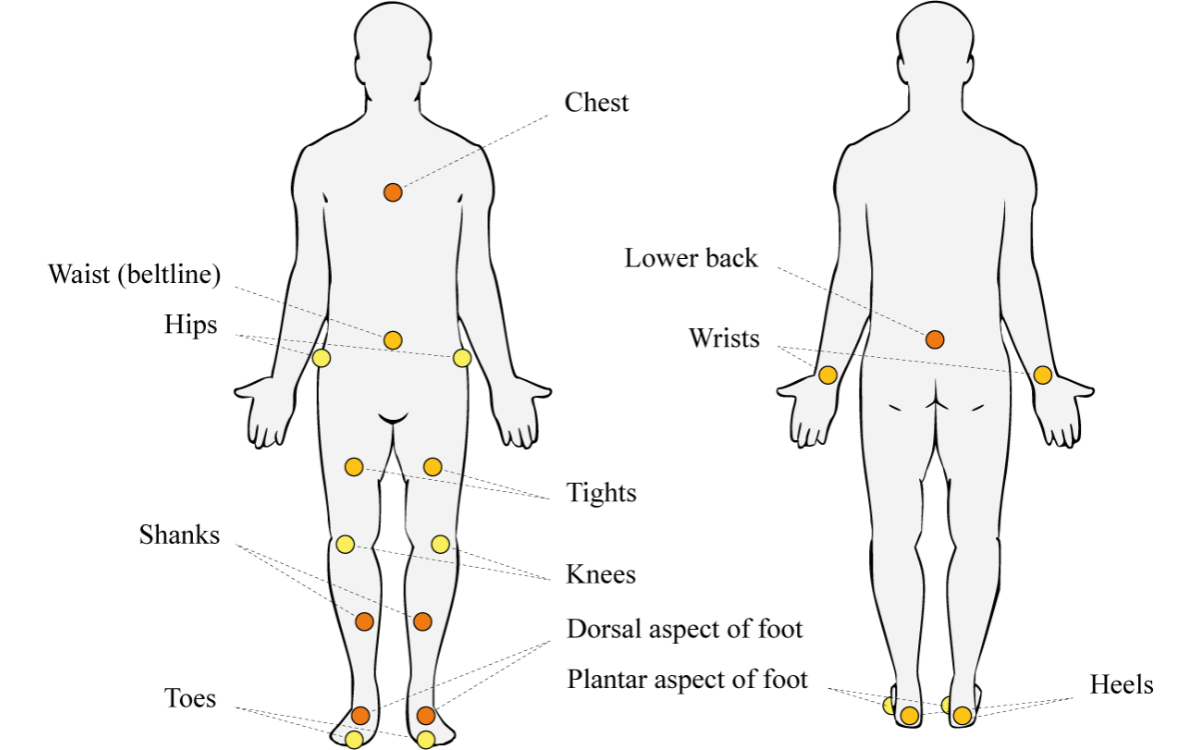
Sensor locations used across the included studies. Marker colors indicate the frequency of use—yellow: n≤2; light orange: n=3-5; and dark orange: n≥6.

The distribution of sensors reveals a concentration on the lower limbs and trunk (Figure 3).

## Discussion

### Principal Findings

In this systematic review, we emphasize the important role of wearable technologies in performing objective and quantitative evaluations of fall risk among patients with neurological disorders. While the studies included were generally well conducted, we noted considerable variability in sensor placement, the types of wearable sensors, motor tasks, and assessment methodologies. In addition, the lack of a standardized approach to fall risk assessment using wearable sensors likely reflects the field’s ongoing technological evolution. To the best of our knowledge, this is the first systematic review analyzing the biomechanical features detected from wearable sensors for fall risk assessment in patients with PD, MS, AD, or stroke. Other studies [21] have investigated the role of inertial sensor–based and insole-based wearable sensors to evaluate fall risk in older adults. In line with our results, these studies identified several biomechanical gait parameters, including spatiotemporal parameters, balance features (center of pressure trajectory), and ADLs that were registered to detect fall risk. However, in our systematic review, we found some differences in the assessment of the risk of falling related mainly to the characteristics of the neurological disorders considered. Furthermore, we noticed differences between the studies concerning technological equipment.

Moreover, another systematic review [56] examined the use of wearable sensors to detect freezing of gait and falls in patients with PD. Consistent with our findings, the authors identified the most common sensor placements, such as the thigh, ankle, and lower back, which align with those reported in our review (Figure 3). However, our study presents some key differences: we included a broader range of neurological disorders (eg, MS, AD, and stroke), focused specifically on fall detection and fall risk assessment, and incorporated more recent literature.

### Pathological and Biomechanical Gait Characteristics Leading to Falls

Falls are very common in individuals with neurological disorders and are influenced by various aspects and features of gait. According to Zhou et al [57], gait variables related to pace (eg, stride and velocity), rhythm (eg, stride duration as well as stance and swing time), variability (eg, SD of ankle dorsiflexion at heel strike), and spatial parameters (eg, stride length, plantar flexion at toe-off, and ankle dorsiflexion at heel strike) can help classify individuals with neurological disorders as fallers. However, neurological disorders are heterogeneous, and each condition is associated with specific gait features that could lead to an increased fall risk; for instance, in patients with MS, although the location, number, and size of MS lesions vary among individuals, certain common characteristics in gait deficits associated with MS have been observed. Patients with MS [58] may exhibit shorter steps, reduced cadence, prolonged double support time, and increased swing phase time. Moreover, some studies have emphasized that patients with MS have reduced joint motion at the hip and ankle and increased joint motion at the knee, with a hyperextension that is more pronounced during midstance [59]. Falls in this patient population can occur early after disease onset; and some factors increase the risk, such as poor visual acuity, impaired postural control, and altered proprioception. However, the studies included in this systematic review analyzed only specific biomechanical metrics related to clinical tests (eg, 30CST) [23,41] or analyzed spatiotemporal features of gait and turning angle [37,44,52]. According to Tulipani et al [41], the 30CST could be a useful test to discriminate “fallers” and “nonfallers” in patients with MS, as it can detect balance impairments and muscle fatigue, both of which are associated with falls. Another important aspect is that remote fall risk monitoring using wearable sensors has the potential advantage of augmenting clinical visits to gain a broader perspective of patient function, symptom fluctuation, and fall risk. In patients with PD, a critical factor that is associated with falls is freezing of gait. However, it is not the only risk factor involved in falls. People with PD can experience bradykinesia, muscle weakness, and body rigidity, all of which contribute to slower gait and are closely linked to fall rates [60]. According to Pelicioni et al [61], reduced quadriceps strength, poor balance and postural stability, and reduced mobility (as indicated by slower TUG test performance) could explain the increased prevalence of balance-related falls. These results are in line with those reported in the included studies; for example, Greene et al [46] found strong associations between fall counts and several individual gait and mobility parameters, including gait variability, during the TUG test. Gait variability refers to fluctuations in gait patterns, and in patients with PD, it reflects a loss of consistency in the ability to produce a steady gait rhythm, thus presenting a likelihood of a fall. In addition, Ma et al [45] found that patients with PD who experienced falls exhibited greater range of motion of the trunk in the sagittal plane than nonfallers. This aspect was identified by the authors as an independent risk factor for falls in patients with PD; and it could be used to predict falls, especially when it is combined with age, gender, and other gait parameters. From these results, it seems that kinematic gait parameters are accurate in detecting fall risk, as also confirmed by Sturchio et al [39]. The authors found that kinematic data extracted from wearable sensors showed greater predictive accuracy for falls than the Hoehn and Yahr scale, which is considered the most robust clinical predictor of falls in individuals with PD. Regarding patients with AD, Huang et al [50] reported that impaired memory is strongly associated with an increased fall risk in individuals with MCI and AD. Specifically, the authors identified TUG time, stride speed, and heel-strike angles as effective parameters for assessing fall risk in this population. However, our review identified only a single study addressing this critical aspect, highlighting a clear gap in the literature. Future research should further investigate this area to determine the most relevant biomechanical and cognitive features for fall risk assessment in patients with AD. Differently from neurodegenerative disorders (eg, MS, PD, and AD), in patients with stroke, both balance and gait deficits related to falls were observed. According to Weerdesteyn et al [62], stroke-related balance deficits include postural instability and lack of coordination in response to both self-induced and external balance perturbations. Gait deficits related to fall risk include reduced propulsion at push-off, decreased hip and knee flexion during the swing phase, and reduced stability during the stance phase. However, the selected studies [36,38] did not report specific biomechanical features related to falls in patients with stroke. In future studies, it could be essential to select and extract specific biomechanical features related to falls in this patient population.

### Technological Equipment: Considerations and Limitations

Fall risk assessment and fall detection have been investigated using wearable technologies. Among these, accelerometers, IMUs, and insole-based systems were mostly used in the clinical context; for instance, triaxial accelerometers and inertial sensors were widely used in the included studies [24]. They measure body movement by detecting speed changes and calculating the resultant acceleration from the displacement of a mass element. In rehabilitation research, the most commonly used types of accelerometers include strain gauge, capacitive, piezoresistive, and piezoelectric sensors. These accelerometers typically have 1 to 3 sensing axes, enabling motion detection in 1D, 2D, or 3D. From our literature analysis [23,37,41,44,51,52], it emerged that triaxial accelerometers were combined with gyroscopes and EMG sensors to capture a wide range of motion data. In particular, gyroscopes calculate changes in angular motion by detecting Coriolis forces, which are proportional to the rate of angular rotation of the limb. Integrating accelerometer and EMG data from the lower limb provides more insightful findings, especially in the context of neurorehabilitation; for example, Meyer et al [48] collected EMG, accelerometer, and gyroscope data that allowed the investigation of foot drop, which has been identified as one of the most frequent causes of falls in individuals with MS. Interestingly, Tulipani et al [41] found significant associations between accelerometer-derived metrics and clinical assessments, notably in balance confidence and fatigue. These findings indicate that wearable sensors can offer a real-time, objective measure of fall risk that aligns well with traditional clinical evaluations. Moreover, we found that the studies on fall risk assessment in PD (10/19, 53%) primarily focused on integrating advanced sensor technologies and complex data analysis methods to monitor, predict, and manage fall risks in patients with PD. Some of these studies (2/19, 11%) used a full-body wearable motion sensor system consisting of 6 sensors (each including a triaxial accelerometer, a gyroscope, and a magnetometer) to detect kinematic gait data. By contrast, Silva de Lima [63] developed a triaxial accelerometer combined with a barometer, which automatically detected falls and allowed users to manually report falls via a button press. This dual functionality enabled the collection of accurate real-time data on fall events.

According to a previous review by Ferreira et al [64], studies involving wearable sensors for fall risk assessment mainly used 1 accelerometer, which underlines the importance of the use of acceleration data in interpreting scores from standard clinical scales. Data from the wearable sensors were used to detect prefall and unbalanced situations, enabling the identification of fall risk events. This approach aims to reduce short-term fall risk by allowing real-time daily monitoring of participants and providing immediate feedback when a fall risk event is detected.

In this context, some of the studies (7/19, 37%) performed the fall risk assessment in an unsupervised setting. Compared to laboratory settings, real-time fall risk prediction during ADLs is more suitable for detecting fall risk events. This approach allows continuous monitoring and enables alert systems to notify participants when fall risk events are detected. However, Meyer et al [48] found that the best performance of the ML model used to predict fall risk was achieved with laboratory data, indicating that controlled environments yield more reliable data for assessing fall risk. Supporting this, some studies have found that specific biomechanical features of gait (ie, speed) can vary depending on whether they are analyzed in the home or laboratory setting; for instance, Carcreff et al [65] found that children with cerebral palsy exhibited lower gait speeds in daily life compared to laboratory settings, potentially due to walking barefoot during laboratory-based assessments. Other studies have also observed discrepancies between supervised and unsupervised gait speeds in older adults. Takayanagi et al [66] identified a weak correlation between daily life and laboratory gait speeds, with daily speeds being significantly lower. De la Cámara et al [67] noted that clinical gait speed measurements might not accurately reflect habitual gait speeds and are influenced by physical, mental, and cognitive health. These studies, which used IMUs, faced limitations such as short walking distances in laboratory settings (2.44-10 meters), which do not adequately represent real-world gait. Even in PD, laboratory assessments do not always mirror daily activity accurately. Toosizadeh et al [68] found no significant correlation between laboratory and home gait parameters, possibly due to small sample sizes and methodological differences. Corrà et al [69] found that certain laboratory tests can better represent gait speed measured at home. However, the ability to remotely monitor patients at home is beneficial for clinicians because it allows them to assess patients’ motor capacity in their domestic environment. In this sense, both in-laboratory and daily living assessments using wearable sensors can provide complementary insights into PD, enhancing clinical evaluations and patient management.

The sensitivity of supervised versus unsupervised assessments for detecting motor fluctuations in PD remains unclear. Therefore, to enhance clinical care and design personalized interventions, a better understanding of gait disabilities is needed, particularly through comprehensive daily life performance data from IMUs.

Although wearable sensors have shown considerable potential in monitoring and detecting fall risk, it is important to acknowledge their limitations and the challenges associated with their use. One critical point is that predicting a fall is not equivalent to preventing it. Current systems are largely reactive, aiming to detect gait abnormalities or balance impairments before a fall occurs, but they do not directly intervene to prevent the event itself. This distinction is crucial, especially in the context of a rapidly aging global population, where fall-related injuries are expected to increase substantially. Therefore, future research should focus not only on improving prediction accuracy but also on integrating real-time intervention mechanisms (eg, alerts, assistive devices, or environmental modifications) that could help prevent falls in a practical setting [21].

Another key challenge lies in ergonomics and user acceptance of wearable technologies. Many current devices lack design considerations for comfort and aesthetics, which can lead to discomfort, skin irritation, or reluctance to use the device, particularly among older adults. These limitations negatively affect long-term adherence and reduce the ecological validity of continuous monitoring solutions. Addressing this issue requires a multidisciplinary approach involving engineers, designers, and end users to codevelop devices that are lightweight and easy to wear. In parallel, user-centered studies should investigate how different sensor placements, materials, and feedback systems influence usability and compliance. By overcoming these ergonomic barriers, wearable technologies can move closer to becoming a truly accessible and effective tool for fall risk management in real-life settings [70].

It is also important to note that data loss and signal noise can affect the reliability of sensor-derived features, especially in real-world settings. To mitigate these issues, careful attention must be given to preprocessing strategies and to the design of data acquisition protocols that minimize artefacts and signal interruptions. Moreover, differences in device types, sensor configurations, and data processing pipelines across studies make cross-study comparisons challenging. Standardization efforts and robust preprocessing techniques are essential to enhance the reproducibility and generalizability of the findings.

### ML Algorithms and Data Processing

In the studies included in this systematic review, a range of AI techniques were applied to analyze data collected via wearable sensors for assessing fall risk in patients with neurological disorders. DL models were prominently featured [37,43,48]. These models are particularly proficient at processing sequential data and capturing temporal dependencies essential for interpreting complex gait patterns over time. This choice reflects a growing trend of using advanced AI methods to detect the complex patterns of movement disorders that are often overlooked by standard clinical assessments. Besides DL, traditional ML techniques also played a significant role; for instance, feature-based models such as support vector machines or random forests were used to classify patients based on the extracted biomechanical features, such as stride length and turning behavior [49,51,52]. Another crucial aspect identified in the included studies was the preprocessing of sensor data using advanced data fusion and signal processing techniques. These methods prepare the raw data for more effective AI analysis by enhancing data quality and extracting meaningful features. Arpan et al [44] used unscented Kalman filter techniques to integrate data from multiple sensors, facilitating a more accurate AI-based prediction of fall events [44]. Some of the included studies (3/19, 16%) explored the implementation of AI models that operate in real time to provide immediate insights or predictive analytics. This approach is particularly useful in a home monitoring context where immediate feedback can prevent falls [63].

Looking ahead, the integration of AI in fall risk assessment could expand in several directions. Tailoring AI models to individual patients’ data over time to adapt to changes in their condition or progression of their neurological disease could improve ongoing monitoring and therapeutic accuracy. By incorporating methodologies that adjust to patient-specific needs and conditions, AI can facilitate more dynamic interventions tailored to the evolving profiles of patients. In a recent study, Espinoza Bernal et al [71] used personalized ML algorithms to monitor and adjust individual rehabilitation strategies for patients with stroke. Using data from wearable sensors during a rehabilitation camp, these algorithms provided high classification accuracy and insights into changes in patient mobility. This capability not only demonstrates the potential of AI to adapt to and predict the needs of patients based on real-time data but also highlights its value in managing conditions that affect mobility and stability, enhancing the precision of therapy and potentially improving patient outcomes [71]. Furthermore, another recent study described neural network models specifically designed to capture the dynamic properties of gait, considering both short-term and long-term dependencies in gait cycles [72]. This approach allowed for the identification of individual-specific gait signatures, which are particularly useful in understanding and accommodating the unique gait dynamics of survivors of stroke, who often exhibit significant variability in their walking patterns due to neurological impairments [72]. The personalization of AI models to adapt to individual patient profiles over time promises to enhance the precision of interventions, although as AI models evolve, there is also a growing need for explainable AI that can make AI decisions more transparent and understandable to clinicians. This will be crucial for building trust and facilitating the integration of AI tools into everyday clinical practice. Future advancements should aim to not only refine the predictive accuracy of fall risk but also ensure that the outcome data are accessible and interpretable for all users involved in the care process. A recent study demonstrated the use of wearable sensors coupled with explainable AI techniques to provide detailed insights into muscle activity changes, aiding clinicians in understanding the predictive outcomes of AI models [73].

### Clinical Considerations and Future Perspectives

Detecting fall risk in individuals with neurological disorders has a primary role in the rehabilitation context. In this review, we identified several biomechanical parameters related to fall risk, as measured by wearable sensors. Data extracted from sensors can provide valuable insights into motor deficits associated with fall risk and guide the development of personalized fall prevention programs. Of note, conventional clinical scales may be not able to capture specific fall-related movements detected by wearable sensors [74]. This discrepancy highlights the importance of body-worn sensors, combining daily life sensor data with clinical assessment outcomes to enhance fall prediction accuracy. Therefore, daily life data recording should be preferred over in-laboratory data collection, as individuals with neurological disorders are more susceptible to the effects of test instructions or external distractions in controlled settings. However, lower activity levels in individuals with neurological disorders may affect the quality of the data collected. Moreover, both the location and method of sensor attachment are crucial when collecting sensor-based data from individuals with neurological disorders. Using multiple sensors might provide more detailed information but is less practical for this patient group. In addition, researchers or trained staff need to be present to ensure the correct placement and wearing of the sensors. From a practical standpoint, the sensor’s location should be chosen carefully to minimize discomfort during the assessment and avoid interfering with natural movement. Technical considerations, such as battery life, data transmission, and storage capacity, must also be factored in when selecting an appropriate sensor for research or clinical practice.

Future studies should incorporate additional fall risk factors (ie, cognitive status, comorbidities, and sensory functions) that could improve the accuracy of fall risk prediction. They should also consider using multiple sensors to capture more detailed biomechanical information. Innovative technologies such as wearable sensors hold promise for enhancing motion analysis and fall risk prediction due to their increasing simplicity, speed, and ease of interpretation compared to traditional methods. However, there remains a scarcity of studies with practical clinical applications for gait analysis in populations with neurological disorders, particularly in patients with stroke or AD. This gap may be attributed to practical challenges, such as the lack of widely available specialized devices in hospital settings and economic constraints. In addition, the absence of direct collaboration between clinicians and bioengineering professionals contributes to this issue. Increased collaboration between physiotherapists, kinesiologists, and bioengineers is essential for sharing practical insights that inform the development of specific motor training programs based on functional assessments. Technological advancements could address these challenges by promoting the use of smart tools, such as smartphones, which are becoming more prevalent in clinical settings, as supported by some studies [13,75,76]. In parallel, advancements in wearable sensor technology are being complemented by the rapid evolution of AI, particularly through its integration with big data, cloud computing, and Internet of Things platforms. Wearable sensors stand to benefit significantly from these advancements, as AI algorithms enable the efficient processing of large and complex datasets; the extraction of informative features; and the generation of accurate, data-driven decisions. These capabilities extend to a range of functions, including gesture recognition, movement analysis, object detection, and continuous health monitoring [77]. Moreover, the integration of multimodal sensors to collect various data types such as motion, physiological, and environmental signals offers promising opportunities for enhancing fall risk assessment. While traditional data fusion methods often struggle with the complexity and heterogeneity of such data, DL provides powerful tools to automatically extract and model relationships across multiple sensor modalities [78].

### Limitations

This systematic review has some limitations that need to be acknowledged. One limitation is the absence of quantitative analysis. In particular, we found considerable heterogeneity among the included studies in terms of sensors used, sensor placement, and biomechanical features extracted; therefore, conducting a quantitative analysis was not feasible. This limitation underscores the need for caution in generalizing the findings and emphasizes the importance of interpreting the results within the context of individual study characteristics. The selected studies also presented other limitations, including small sample sizes, lack of a control group or normative data, and lack of long-term follow-up evaluations. Future large-scale longitudinal studies are warranted to further explore and validate key aspects of fall risk assessment and fall detection in patients with neurological disorders.

Despite these limitations, our review provides a comprehensive qualitative synthesis that systematically summarized and interpreted the findings of individual studies to elucidate common themes, patterns, and discrepancies in the literature. As such, it offers valuable insights into the biomechanical features and wearable sensors used to perform fall risk assessment, identifying key implications for clinical practice and considerations for future investigation.

### Conclusions

In this systematic review, we highlighted the critical role of wearable technologies in providing an objective and quantitative assessment of fall risk in patients with neurological disorders. Although the included studies were well executed, we identified significant heterogeneity in sensor placement, types of wearable systems, motor tasks, and evaluation methods. In addition, we observed a tendency among the included studies to primarily focus on technological and engineering aspects, often at the expense of the clinical context. However, the absence of a standardized fall risk assessment using wearable systems is likely attributable to ongoing technological advancements, which can be considered a strength.

## Appendix

Multimedia Appendix 1 PRISMA (Preferred Reporting Items for Systematic Reviews and Meta-Analyses) checklist.

Multimedia Appendix 2 Search queries.

## Abbreviations

30CST: 30-second chair stand test
ADL: activity of daily living
AI: artificial intelligence
AUC: area under the receiver operating characteristic curve
DL: deep learning
EMG: electromyography
IMU: inertial measurement unit
LSTM: long short-term memory
ML: machine learning
MS: multiple sclerosis
PD: Parkinson disease
PRISMA: Preferred Reporting Items for Systematic Reviews and Meta-Analyses
TUG: timed up and go
