# Supplementary material for: Use of Wearable Sensors to Assess Fall Risk in Neurological Disorders: Systematic Review
Source: JMIR Mhealth Uhealth. 2025 Aug 18;13:e67265. doi: 10.2196/67265 (PMC12402735; doi:10.2196/67265)
Supplement: Multimedia Appendix 2 [file mhealth_v13i1e67265_app2.docx]

**Search queries**

*Pubmed*

((fall[Title]) AND (parkinson[Title/Abstract] OR 'multiple sclerosis'[Title/Abstract] OR stroke[Title/Abstract] OR 'traumatic brain injury'[Title/Abstract] OR alzheimer[Title/Abstract])) AND (wearable[Title/Abstract])

*Web of Science*

TI=fall AND (TS=(parkinson) OR TS=(multiple sclerosis) OR TS=(stroke) OR TS=(traumatic brain injury) OR TS=(alzheimer)) AND TS=wearable

*Embase*

('fall'):ti AND ((parkinson):ti,ab OR (multiple sclerosis):ti,ab OR (stroke):ti,ab OR (traumatic brain injury):ti,ab OR (alzheimer):ti,ab) AND (wearable):ti,ab

*IEEE Xplore*

("Document Title":fall) AND ((("Document Title":parkinson) OR ("Abstract":parkinson)) OR (("Document Title":multiple sclerosis) OR ("Abstract":multiple sclerosis)) OR (("Document Title":stroke) OR ("Abstract":stroke)) OR (("Document Title":traumatic brain injury) OR ("Abstract":traumatic brain injury)) OR (("Document Title":alzheimer) OR ("Abstract":alzheimer))) AND (("Document Title":wearable) OR ("Abstract":wearable))
